# Two Cases of Rubinstein-Taybi Syndrome With Retinal Detachment

**DOI:** 10.7759/cureus.80048

**Published:** 2025-03-04

**Authors:** Natsumi Kawaguchi, Fukutaro Mano, Hiroyuki Kondo, Kazuki Kuniyoshi, Shunij Kusaka

**Affiliations:** 1 Department of Ophthalmology, Kindai University Hospital, Osakasayama, JPN; 2 Department of Ophthalmology, University of Occupational and Environmental Health, Kitakyushu, JPN

**Keywords:** familial exudative vitreoretinopathy (fevr), neovascular glaucoma, pars plana vitrectomy, retinal detachment (rd), rubinstein-taybi syndrome (rsts)

## Abstract

We report two cases of Rubinstein-Taybi syndrome (RTS) with retinal detachment. Case 1 is a nine-year-old girl with RTS. She developed a macula-involving tractional retinal detachment (TRD) in the superior temporal quadrant of her right eye. Lens aspiration, intraocular lens implantation, vitrectomy with membrane peeling, and encircling were performed. However, retinal reattachment was not achieved. Two months after the surgery, neovascular glaucoma (NVG) in the right eye developed and consequently became phthisis bulbi two years after the surgery. At the age of 13 years, she underwent cataract surgery and vitrectomy for mature cataracts and worsening TRD in the left eye. Eleven months after surgery, NVG developed in the left eye, which was refractory to multiple glaucoma surgeries. In addition, the retina was re-detached and could not be reattached due to severe proliferative vitreoretinopathy. Case 2 is a 15-year-old boy with RTS, which was diagnosed immediately after birth. At the age of 10 years, total retinal detachment with a horseshoe tear occurred in the inferior temporal quadrant in the left eye, which underwent lensectomy, vitrectomy, and silicone oil tamponade. To achieve retinal reattachment, three vitrectomies were required. However, secondary angle-closure glaucoma developed one year after the last surgery. The intraocular pressure was poorly controlled even after trabeculotomy and goniosynechialysis, and his vision became no light perception three years postoperatively. Retinal detachment in patients with RTS may be challenging to treat, and multiple surgeries are required. Special caution is exercised regarding postoperative refractory glaucoma.

## Introduction

Rubinstein-Taybi syndrome (RTS) is a congenital anomaly named after Drs. Rubinstein and Taybi, who described seven patients who exhibited intellectual disability, dysmorphic facial features, and broad thumbs and toes in 1963 [1]. RTS is an extremely rare disease, with a prevalence of one in 100,000-125,000 births [2]. The cAMP response element-binding protein binding protein (CREBBP) gene located on the short arm of chromosome 16 (16p13.3) and the adenovirus early region 1A-associated protein P300 (EP300) are so far identified as responsible genes [3,4]. Approximately 60% and 8% of patients with RTS have CREBBP and EP300 mutations, respectively [3,4]. Thus, clinical findings are of diagnostic importance [5]. Although most RTS cases are sporadic, it can be transmitted in an autosomal dominant mode. It is unknown whether the life span of RTS is abnormal. However, over 90% of individuals with RTS are reported to survive into adulthood [5]. Multidisciplinary care by specialists to improve quality of life and reduce complications is recommended.

The primary clinical characteristics of RTS include intellectual disability, growth retardation, broad thumbs and toes, and dysmorphic facial features [1]. Regarding ocular complications, nasolacrimal duct obstruction, high myopia, strabismus, developmental glaucoma, congenital cataract, and choroidal coloboma have been described [6,7].

Retinal detachment appears to be a rare complication of RTS. To the best of our knowledge, only one case of RTS with rhegmatogenous retinal detachment has been reported to date [8]. Herein, we report two cases of RTS, namely, tractional retinal detachment (TRD) in case 1 and rhegmatogenous retinal detachment in case 2.

## Case presentation

Case 1

She was born in early-term (gestational age of 37 weeks six days; birth weight was 1966 g) and clinically diagnosed with RTS immediately after birth based on the features of broad thumbs and toes and dysmorphic facial abnormalities. Chromosomal microarray analysis revealed a heterozygous deletion of all CREBBP regions (arr[hg19] 16p13.3(3657088_6552944)X1). She underwent surgical treatment for vascular rings, patent ductus arteriosus, ventricular septal defects, and duodenal atresia immediately after birth. She also had severe intellectual disability. Her family history was unremarkable.

At the age of nine years, she was referred to our clinic because of decreased vision. A cataract was observed in her right eye. Further ophthalmoscopic fundus examination revealed TRD with fibrovascular proliferation in the superior temporal quadrants in both eyes. In the right eye, TRD seemed to be progressing and threatening the macula. Thus, lensectomy, intraocular lens (IOL) implantation, vitrectomy with membrane peeling, and encircling (#240) were performed for the right eye. However, retinal reattachment was not achieved because of the development of severe proliferative vitreoretinopathy (PVR). Hence, the detached retina in the left eye was carefully monitored because the lesion did not involve the posterior pole. Two months after the surgery for the right eye, neovascular glaucoma (NVG) developed. Cyclocryopexy was required, and the intraocular pressure was reduced successfully. At that time, her best-corrected visual acuity (BCVA) was no light perception in the right eye and 20 cm/CF in the left eye. The intraocular pressures were 8 and 15 mmHg in the right and left eyes, respectively.

**Figure 1:**
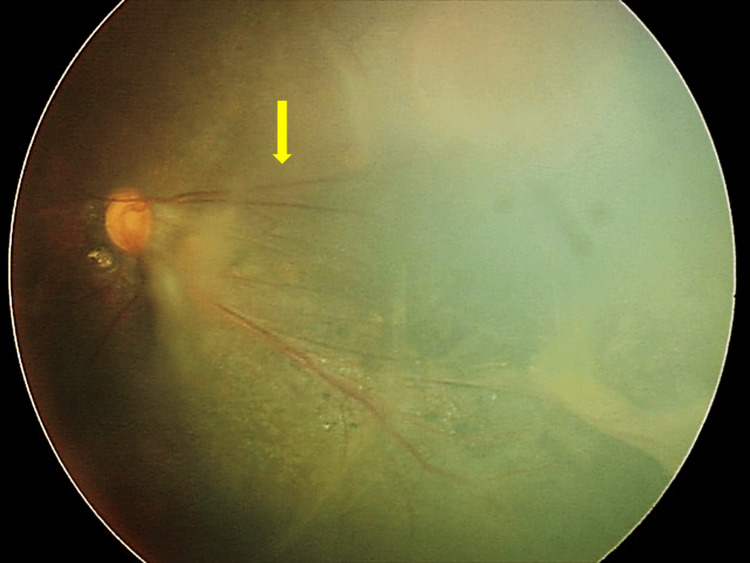
Preoperative fundus photograph of case 1 Tractional detachment was observed in the superior temporal quadrant and the straightening of the vessels toward the peripheral retina resembled familial exudative vitreoretinopathy.

At the age of 13 years, a mature cataract developed, and the fundus view was obscured in the left eye. As worsening of TRD was suspected by B-scan ultrasonography, lens aspiration, IOL implantation, and vitrectomy were performed. During the vitrectomy, membrane peeling for the peripheral proliferative tissue was performed, followed by a laser photocoagulation on the ischemic retina. During these procedures, straightening of the vessels toward the peripheral retina, which seemed similar to familial exudative vitreoretinopathy (FEVR), was noted (Figure 1).

At the age of 14 years (11 months after the initial vitrectomy), NVG developed in the left eye, and the intraocular pressure was 35 mmHg. The intraocular pressure was poorly controlled even after multiple glaucoma surgeries (trabeculotomy and Ahmed valve implantation). TRD in the left eye remained stable for two years after the initial vitrectomy. However, two years after the initial vitrectomy, hyphema occurred, and a vitrectomy (fifth surgery) was performed. After cleaning the intraocular hemorrhage, PVR was noted with new retinal tears located in the inferior and superior nasal quadrants. Retinal reattachment could not be achieved because of the severe PVR.

Case 2

He was born in full term. He was clinically diagnosed with RTS soon after birth based on dysmorphic facial features, broad thumbs, and broad toes. He underwent surgery for cryptorchidism in his infancy. He had intellectual disability. His family history was unremarkable. He regularly visited a local ophthalmology clinic for myopia, amblyopia, and entropion of both eyes. At the age of five years, he underwent surgery for entropion in the left eye. At that period, his BCVA was 0.4 (20/50) in both eyes.

At the age of 10 years, he was referred because of decreased vision in the left eye.

**Figure 2:**
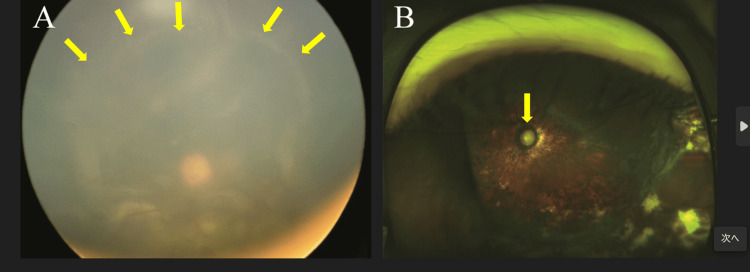
Pre- and postoperative fundus photograph of the left eye of case 2 (A) Total retinal detachment with a retinal tear on the inferior temporal side was observed. (B) Retinal reattachment was achieved; however, the glaucomatous optic neuropathy resulted in the final visual acuity of no light perception.

**Figure 3:**
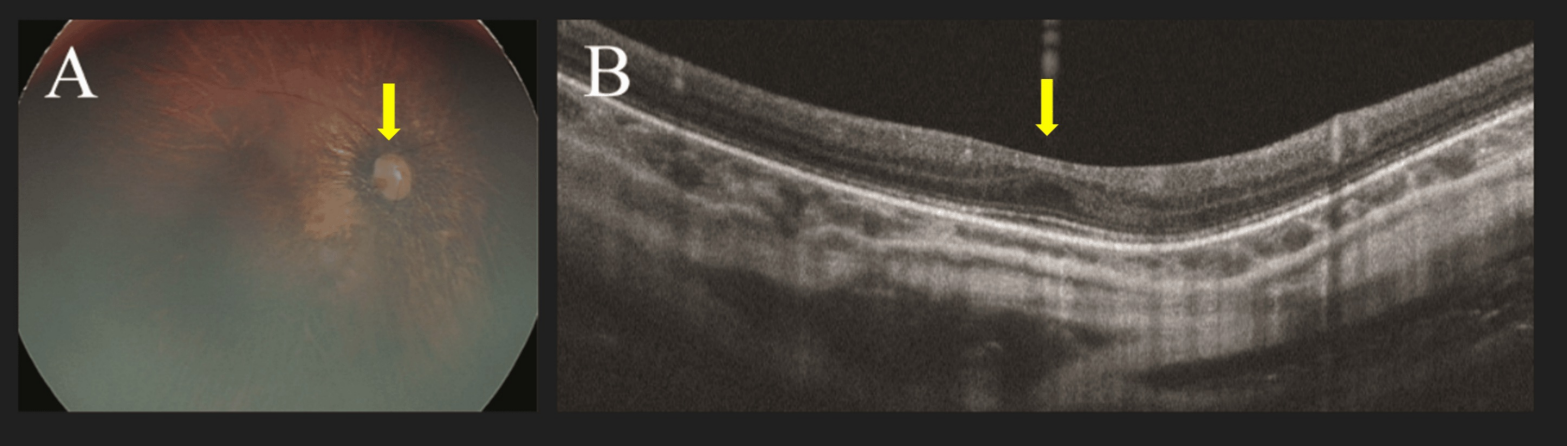
Fundus photograph and optical coherence tomography image of the right eye of case 2 (A) Excavated optic disc and macular reflex loss was observed. (B) Foveal hypoplasia as foveal excavation was not observed.

His visual acuities were 0.1 (20/200) and 0.01(20/2000) in the right and left eyes, respectively. Fundus examination revealed total retinal detachment with a horseshoe tear in the inferior temporal quadrant in the left eye (Figure 2). A fundus examination in the right eye revealed an excavated optic disc and foveal hypoplasia (Figure 3).

He underwent lens aspiration, vitrectomy, and silicone oil tamponade in the left eye. Two months after the initial surgery, the retina was redetached, and encircling scleral buckling (#277) and silicone oil tamponade were performed. Three months after the second surgery, the silicone oil was removed. Five months after silicone oil removal, secondary angle-closure glaucoma developed, and the intraocular pressure was poorly controlled even after trabeculotomy and goniosynechialysis. His visual acuity was light perception by three years after the last surgery and became no light perception thereafter. Because the patient had poor vision, further glaucoma intervention was not performed.

## Discussion

Of the two RTS cases reported herein, case 1 had a TRD in both eyes, and case 2 had a rhegmatogenous retinal detachment with the causative tear on the inferior temporal quadrant in the left eye. RD of both cases were refractory and multiple vitrectomies were required. Moreover, secondary glaucoma occurred after surgery in all three eyes, and the intraocular pressure was poorly controlled by intense therapy including multiple anti-glaucoma agents and multiple glaucoma surgeries.

Patients with RTS have diverse eye symptoms, including lacrimal duct obstruction, pupil and iris abnormalities, corneal abnormalities, glaucoma, cataract, and high myopia [6,7]. However, detailed findings of the fundus with RTS are rarely reported [8]. Van Genderen et al. examined the fundi of 24 patients with RTS and found macular abnormalities in 18 (75%). The absence of the foveal reflex and macular degeneration was noted in 13 (54%) and five (21%) cases, respectively [6], and excavated discs were observed in nine (38%) [6]. As indicated by optical coherence tomography, foveal hypoplasia and an excavated optic disc were noted in case 2, as with common features of the abovementioned findings.

A rhegmatogenous retinal detachment was previously reported as a complication in only one case of RTS [8]. The causative tear in this case was located on the temporal side. The preoperative refraction in that case was −12 D, indicating high myopia. Retinal reattachment was achieved by scleral buckling, and the postoperative course was uneventful during the three-month follow-up [8]. RTS is often accompanied by high myopia. In the study by Van Genderen et al., six of 24 (25%) patients with RTS had a myopia of −6D or worse [6]. In case 2, the spherical equivalent in the OD was −17 D, supporting the link between RTS-associated high myopia and retinal detachment.

TRD as a complication of RTS has not been reported previously, and the findings in case 1 might be similar to those of FEVR. Proliferative membrane formation on the ischemic retina and TRD on the superior temporal quadrant, dragged disc, and straightening of vessels were also observed. It remains possible that the patient had a mutation in genes causing FEVR, e.g., FZD4, NDP, LRP5, and TSPAN12. Another differential diagnosis is autosomal dominant neovascular inflammatory vitreoretinopathy (ADNIV), which is a rare inherited autoimmune eye disease characterized by uveitis and retinal detachments and NVG [9]. ADNIV is caused by missense mutations in CAPN5, which is located on chromosome 11q13.5. Such missense mutations are undetectable by the chromosomal microarray analysis. Further study is required to assess the involvement of ADNIV genes.

The postoperative intraocular pressure was poorly controlled in the two cases. In case 1, the final intraocular pressure following multiple glaucoma surgeries (including Ahmed transplantation) was 40 mmHg. In case 2, the final intraocular pressure following trabeculotomy and goniosynechialysis was 30 mmHg. According to Van Genderen, approximately 38% of patients with RTS had congenital glaucoma, particularly with developmental anomalies of the iridocorneal angle [6]. Surgical outcomes for glaucoma with angle dysgenesis are generally unfavorable. A previous study reported three RTS cases with glaucoma treated surgically in their infancy [10]. In one case, a five-month-old boy underwent multiple sessions of trabeculotomy and trabeculectomy in both eyes, without successfully reducing the intraocular pressure. Following a combination of glaucomatous optic neuropathy and the previously treated retinal detachment, the vision status became light perception [10].

In our two cases, vitrectomy and silicone oil tamponade were used to achieve retinal reattachment. Although different glaucoma pathology underlies between cases 1 and 2, congenital abnormality of the angle of RTS is susceptible for postoperative glaucoma. Aphakic status and the use of silicone oil tamponade could also potentially raise risks of secondary angle-closure glaucoma. Caution should be taken for postoperative glaucoma development in RTS cases requiring retinal detachment repair, as indicated in our cases.

## Conclusions

Of the two RTS cases reported herein, case 1 had a TRD in both eyes, and case 2 had a rhegmatogenous retinal detachment. RD of both cases were refractory, and multiple vitrectomies were required. Tractional and rhegmatogenous retinal detachments can complicate RTS, and the prognosis following vitreous surgery in such cases was considered extremely poor. Therefore, caution should be exercised once secondary glaucoma occurs after surgery.
